# 14-3-3ε protein-loaded 3D hydrogels favor osteogenesis

**DOI:** 10.1007/s10856-020-06434-1

**Published:** 2020-11-03

**Authors:** Ana A. Aldana, Marina Uhart, Gustavo A. Abraham, Diego M. Bustos, Aldo R. Boccaccini

**Affiliations:** 1 Instituto de Investigaciones en Ciencia y Tecnología de Materiales, INTEMA (UNMdP-CONICET), Mar del Plata, Argentina; 2 Institute of Biomaterials, Department of Materials Science and Engineering, University of Erlangen-Nuremberg, Erlangen, Germany; 3 Laboratorio de Integración de Señales Celulares, Instituto de Histología y Embriología de Mendoza (IHEM-CONICET-UNCuyo), Mendoza, Argentina; 4 Facultad de Cs Exactas y Naturales -UNCuyo Mendoza, Mendoza, Argentina

## Abstract

3D printing has emerged as vanguard technique of biofabrication to assemble cells, biomaterials and biomolecules in a spatially controlled manner to reproduce native tissues. In this work, gelatin methacrylate (GelMA)/alginate hydrogel scaffolds were obtained by 3D printing and 14-3-3ε protein was encapsulated in the hydrogel to induce osteogenic differentiation of human adipose-derived mesenchymal stem cells (hASC). GelMA/alginate-based grid-like structures were printed and remained stable upon photo-crosslinking. The viscosity of alginate allowed to control the pore size and strand width. A higher viscosity of hydrogel ink enhanced the printing accuracy. Protein-loaded GelMA/alginate-based hydrogel showed a clear induction of the osteogenic differentiation of hASC cells. The results are relevant for future developments of GelMA/alginate for bone tissue engineering given the positive effect of 14-3-3ε protein on both cell adhesion and proliferation.

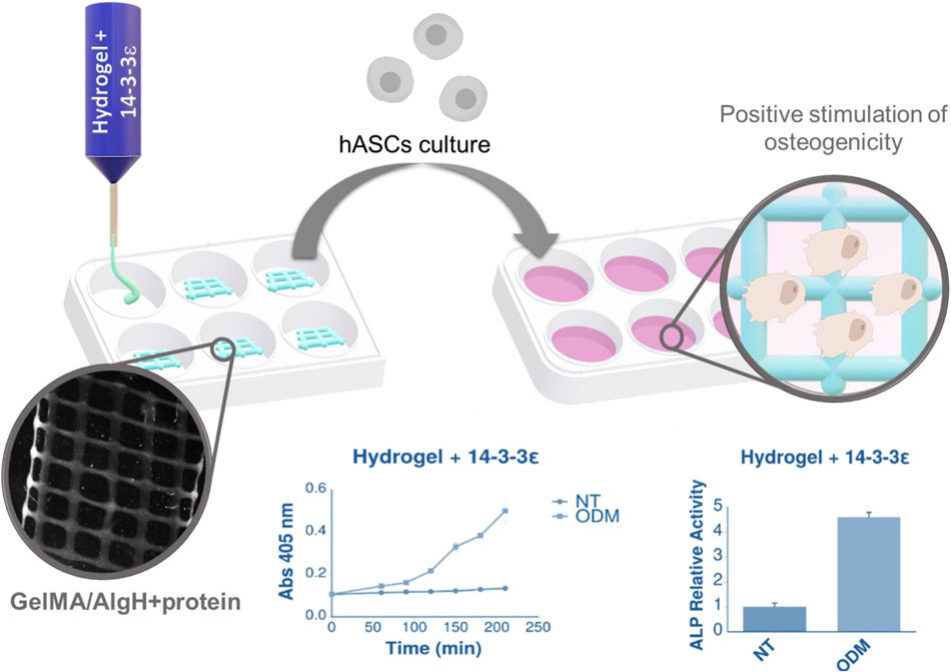

## Introduction

3D printing has emerged as a novel approach capable of assembling cells, biomaterials, and biomolecules in a spatially controlled manner to reproduce native tissue [1, 2]. The biocompatible materials needed for this technique are mainly made of biopolymer-based hydrogels [1–4]. The high-water-content and physical properties of hydrogels can mimic the extracellular matrix. Incorporation of biochemical signals into the printed matrix is a convenient strategy to induce differentiation of stem cells [5]. Particularly, 14-3-3ε protein was recently studied for differentiation of human adipose derived stem cells (hASC) to osteoblasts on PCL-nHA scaffolds [6]. The 14-3-3 protein family includes in mammals seven highly conserved isoforms, involved in a wide range of vital regulatory processes. Under normal conditions, these proteins are intracellular; however, some reports have recognized their presence outside the cell [7]. Recently, 14-3-3ε was identified as a novel soluble mediator between subchondral bone and cartilage, and it was demonstrated that it is specifically released by osteoblasts in response to mechanical stress [8]. Although this protein can bind to CD13, which plays pivotal roles in many physiological processes such as differentiation, proliferation, and adhesion [9], its extracellular functions have not been fully elucidated yet. hASCs cells are promising in the field of regenerative medicine due to the many advantages over other stem cells [10]. The improvement of bone scaffolds by incorporating hASCs to accelerate the osteointegration and to enhance the efficiency in the formation of uniform tissue has been reported [10], being an attractive approach in bone regeneration strategies.

The aim of this work was to develop printed scaffolds based on GelMA-alginate hydrogels encapsulating 14-3-3ε protein, which could stimulate the early stages of osteogenic differentiation of hASCs [6].

## Materials and methods

### Ink preparation

Sodium alginate of low and high viscosity (AlgL 4–12 cps and AlgH 15–25 cps, respectively, Sigma-Aldrich) were dissolved in phosphate buffer (PBS) at 4%wt. and stirred 4 h at 30 °C. GelMA (4%wt.), synthesized as previously reported [11], and photoinitiator (0.5%wt. Irgacure2959) were then added to the AlgL and AlgH solutions and stirred overnight. Thus, the final polymer concentration was 8%wt.

### 3D printing

Each blend was loaded into a syringe at room temperature for use with the BioScaffolder2.1 bioprinter (GeSIM, Großerkmannsdorf, Germany). Grid-like, square structures with an edge length of 15 mm were plotted to generate test specimens. The hydrogels were printed into six well culture plates (VWR, Germany). For the printing of the hydrogel, 200 μm (inner diameter) nozzle, 60–80 kPa pressure and 10 mm s^−1^ plotting speed were set. After processing, ionic crosslinking was performed using 0.1 M CaCl_2_ solution for 10 min and the samples were then washed with deionized H_2_O. Chemical crosslinking was performed by irradiating the hydrogels 10 min at 365 nm (45 W UV-LED array homemade, 4 mW/cm^2^).

### Characterization of printed hydrogels

Hydrogels were observed by bright field microscopy (PrimoVert, Carl Zeiss, Germany). The strand width and pore size were determined from images using ImageJ software (open source). The printing accuracy was calculated comparing the area of the printed structure with the area of the designed structure.

### 14-3-3ε protein recombinant expression in Escherichia coli

The gene of human 14-3-3ε was cloned in the vector pET24a (Clontech), expressed in *Escherichia coli*, and purified from whole lysates by using the C-terminal His6-tag. Purity was checked by SDS-PAGE [12].

### Printing protein-loaded GelMA/AlgH hydrogels

Due to the high printing accuracy, GelMA/AlgH was chosen for protein encapsulating. 14-3-3ε (15 mg) was added to 10 ml GelMA/AlgH solution (Section 2.1) and stirred overnight at room temperature. Then, the protocol of Section 2.2 was followed to obtain printed protein-loaded hydrogel scaffolds.

### Cell culture and ALP activity evaluation

Healthy female patients, 30–40 years old, voluntarily donated abdominal subcutaneous adipose tissue after signing an informed consent in accordance with the Declaration of Helsinki (National University of Cuyo, Bioethics Committee). Material was processed to obtain the hASCs as described in detail by Gojanovich et al. [13]. Briefly, adipose tissue was digested by collagenase type I (Roche) 1 mg/ml at 37 °C for 1 h with mild agitation. After centrifugation of the digested tissue, the pellet containing the stromal vascular fraction (that contained hASCs plus other cells) was seeded in a 10 cm petri dish and cultured in DMEM, 10% FBS, 100 U/ml penicillin/streptomycin under standard conditions (37 °C, 5% CO_2_). After 1 day, hASCs were attached to the plate surface and could be separated from the other cells (non-adherent cells) present in the stromal vascular fraction. Finally, isolated hASCs were characterized by flow cytometer analysis (CD105, CD90, CD73 positive markers, and CD45, CD34, CD11b, CD19, HLA-DR negative markers) and by differentiation to adipogenic and osteogenic lineages [13]. Isolated hASCs were seeded on each previously sterilized (UV light 20 min) scaffold at 40,000 cells/cm^2^ and grown in standard culture conditions (DMEM, 10% FBS, 100 U/ml penicillin/streptomycin, 37 °C, 5% CO_2_) for 3 days. Cell adhesion and proliferation were measured by cell counting in Neubauer chamber. Viability was measured by alkaline phosphatase (ALP) activity assay [14]. Osteogenic differentiation of hASCs was induced with a drug cocktail containing 10 mM β-glycerophosphate, 0.1 µM dexamethasone and 50 µg/mL 2-phospho-L-ascorbic acid [6]. ALP activity was colorimetrically measured (multiskan FC, Thermo Fisher Scientific) by reading the absorption of *p*-nitrophenylphosphate at λ = 405 nm (ε = 18.3 mM ^−1^ cm ^−1^) on fixed (4% *p-*formaldehyde) and permeated cells (phosphate buffer saline, 0.05% Tween-20). The results from six independent experiments were analyzed using one-way analysis of variance (ANOVA, *p* < 0.05).

## Results

**Fig. 1:**
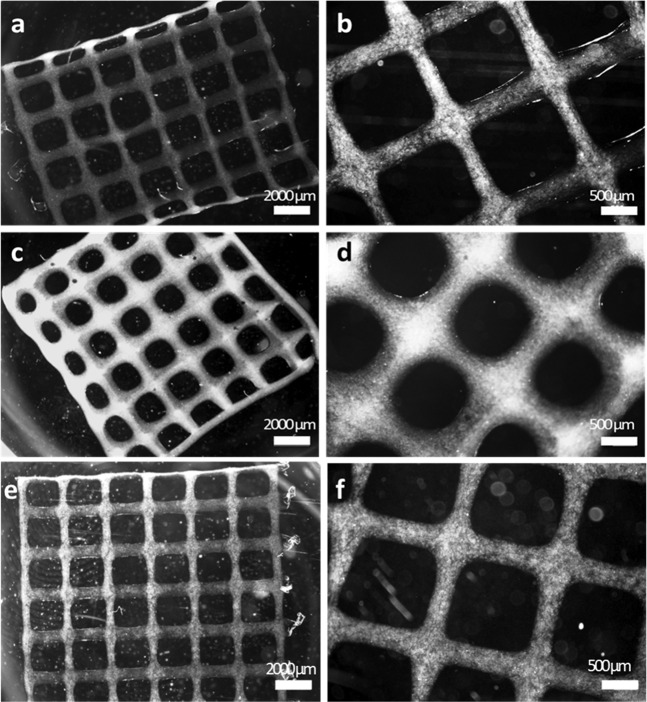
SEM images of printed GelMA/AlgH (**a**, **b**), GelMA/AlgL (**c**, **d**) and GelMA/AlgH+protein (**e**, **f**) hydrogels

3D printed hydrogels have gained growing attention due to their capacity to mimic the complex structure of native human tissue. It has been demonstrated that both physical and biological cues affect cell proliferation and differentiation. In this work, 3D printed hydrogel scaffolds with grid-like structure (GelMA/AlgL and GelMA/AlgH) were developed and characterized. The creation of grid structures provides the possibility of cell immobilization in a defined geometry to form porous scaffolds of tailored shape for improved vascularization. For vascularization a pore size higher than 300 μm is proposed by Fedorovich et al. [15]. 3D print parameters were first optimized to obtain GelMA/AlgH and GelMA/AlgL hydrogels. Optical images of printed hydrogels (Fig. 1) show the structures obtained for both blends. While the strut width of AlgH-based hydrogel (0.46 ± 0.05 mm) was lower than AlgL-based hydrogel (0.80 ± 0.09 mm), the pore size of GelMA/AlgH (3.65 ± 0.50 mm^2^) was higher than that of GelMA/AlgL (2.13 ± 0.34 mm^2^). The printing accuracy also increased by rising the alginate viscosity (from 67 ± 4 to 92 ± 4%). The results demonstrate that printing fidelity of GelMA/AlgH ink is greater than the one of GelMA/AlgL. Physical and chemical crosslinking allowed to avoid hydrogel structure collapse. GelMA/AlgH hydrogel was chosen as ink to print structures encapsulating 14-3-3ε protein.

**Fig. 2:**
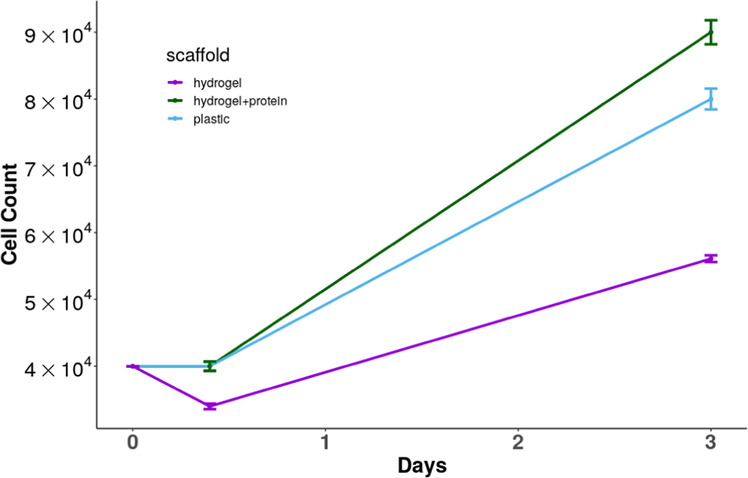
Proliferation of hASC cultured for 3 days on hydrogel or hydrogel+14-3-3e compared to the level of growth in a plastic cell culture plate. Cells were counted using a Neubauer chamber just before seeding them at 40,000 cells/cm^2^ on the scaffold, then they were counted over the hydrogel after 6 h (cell adhesion) and after 3 days of culture (cell proliferation). Data points are means of six independent experiments in duplicate (±SD)

hASCs cells showed their typical fibroblastoid morphology [13] and osteogenic differentiation potential when cultured on all hydrogel scaffolds. The cellular adhesion and proliferation on the hydrogel (GelMA/AlgH) and on the protein-encapsulated hydrogel (GelMA/AlgH+protein) were significantly different. The addition of 14-3-3ε protein to GelMA/AlgH hydrogel had a positive effect on cellular adhesion, increasing from 85.0 ± 1.2% on neat hydrogel (GelMA/AlgH) to 100.0 ± 1.8% on protein-loaded hydrogel. The cell proliferation of hASC on GelMA/AlgH and GelMA/AlgH+protein hydrogels was 65.0 ± 0.8% and 125.0 ± 1.9%, respectively (Fig. 2).

The incorporation of the protein in the hydrogels improved the cell proliferation. Furthermore, the effect of protein addition on hASC proliferation was higher than the proliferation observed on the control (plastic culture plates). In addition, there was no evidence of death cells in any scaffold. The results confirmed the expected positive effect of the protein on hASC proliferation. This strong effect of 14-3-3ε protein in hASC proliferation was previously observed on electrospun scaffolds modified with the same protein [6].

**Fig. 3:**
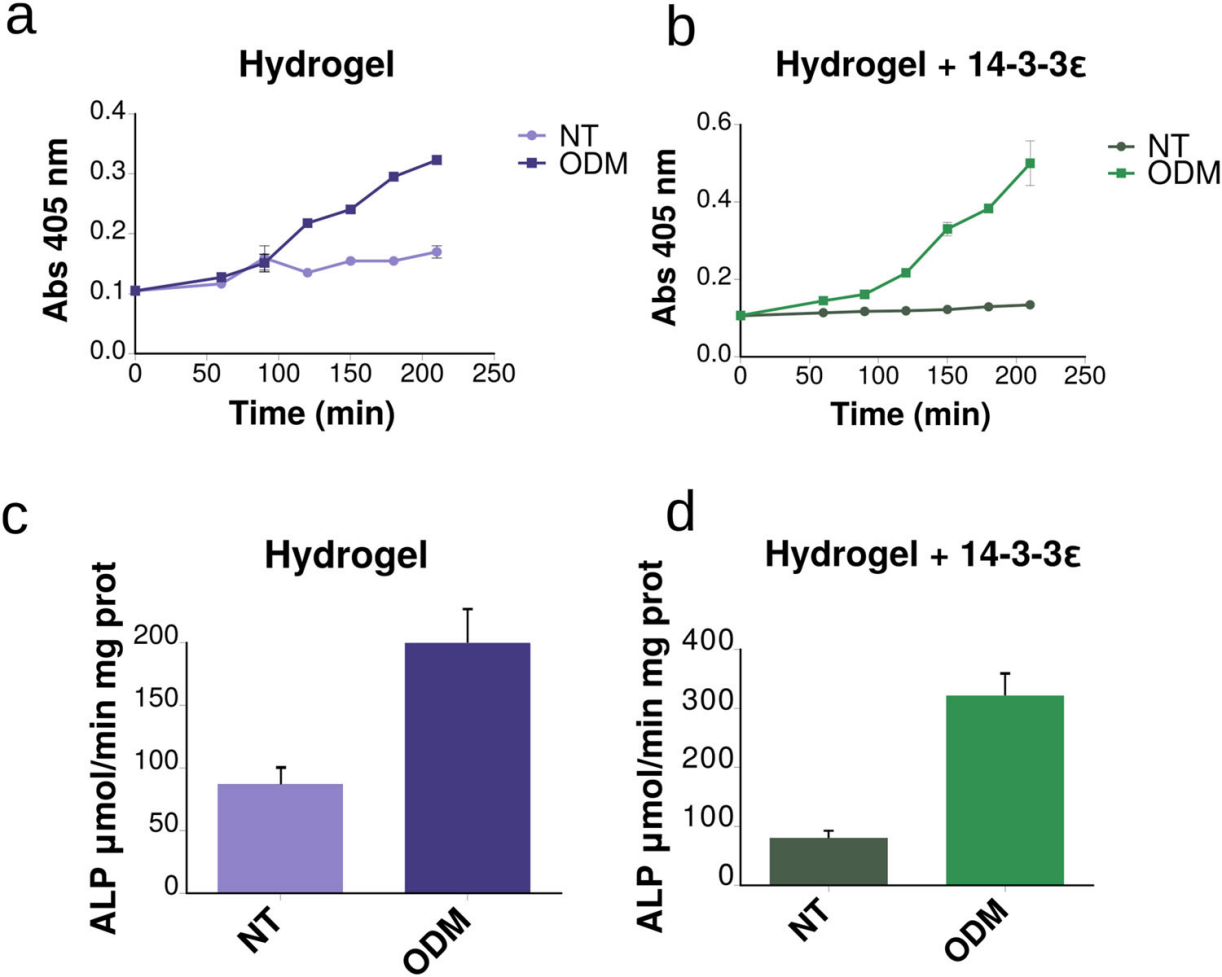
Enzymatic kinetics of the early biomarker of osteogenesis ALP on non-treated and differentiated hASCs on hydrogel scaffolds. Hydrogel (**a**, **c**) and hydrogel+14-3-3ε (**b**, **d**) means GelMA/AlgH and GelMA/AlgH+protein, respectively. After 3 days of growing on either standard DMEM supplemented with 10% fetal bovine serum (NT) or osteogenic differentiation medium (ODM), the activity of ALP was colorimetrically measured through absorbance lecture of its hydrolyzed substrate at 405 nm at different time points (**a**, **b**). Bars in the lower graphs represent the International Units calculated from the average slope of the upper ones normalized by mg of protein. Data points represent the mean of six independent experiments in duplicate (±SD)

In order to observe the effect on hASC differentiation, the activity of ALP was measured. ALP is an early biomarker of the differentiation from mesenchymal cell to pre-osteoblast/mature osteoblast, which is absent in the osteocytes (final stage) [16]. The expression of this biomarker can be induced by osteogenic drugs, and in the present study the hydrogel did not interfered physically nor chemically with the activity of these drugs on hASC. Figure 3 shows the relative activity of ALP in hASCs growing on the hydrogels. The strong effect of the 14-3-3ε recombinant protein on promoting hASCs osteogenic differentiation was evidenced when considering that the hydrogel+protein induced three times more ALP activity than did the neat hydrogels. In our previous work [6], the protein binding on the electrospun fiber surfaces enhanced the ALP activity in PCL-nHA/protein scaffolds which was four times higher than in non-modified PCL-nHA scaffolds, indicating osteogenic stimulation. The protein could be acting as a cytokine, enhancing both cell adhesion and proliferation, consistent with its presence in extracellular vesicles liberated from osteoblasts [17]. The increase of ALP activity measured in the protein-encapsulated hydrogel scaffold highlights the strong promoting effect of this recombinant protein on hASCs osteogenic differentiation. To our knowledge, this is the first report combining the 14-3-3ε protein as a bioactive molecule in 3D printed hydrogel scaffolds, which shows a remarkable effect on hASCs osteogenic differentiation.

## Conclusions

Hydrogel inks based on gelatin methacrylate (GelMA) and alginate were developed successfully. 3D hydrogel scaffolds were printed with high fidelity, and the structure maintained its shape after photo-crosslinking. Human 14-3-3ε recombinant protein was purified and encapsulated in the hydrogel ink. 14-3-3ε protein-loaded GelMA/AlgH hydrogel improved significantly the osteogenic differentiation of hASC. The incorporation of this biological cue is also relevant to stimulate both cell adhesion and proliferation. This work has confirmed that 14-3-3ε could be used as a biological factor to enhance 3D printed biomaterials for bone tissue engineering.

## References

[1] Zhang Y, Yu Y, Akkouch A, Dababneh A, Dolati F, Ozbolat IT (2015). In vitro study of directly bioprinted perfusable vasculature conduits. Biomater Sci.

[2] Dzobo K, Thomford NE, Senthebane DA, Shipanga H, Rowe A, Dandara C (2018). Advances in regenerative medicine and tissue engineering: innovation and transformation of medicine. Stem cells Int.

[3] Vyas D, Udyawar D (2019). A review on current state of art of bioprinting. 3D Printing and Additive Manufacturing Technologies.

[4] He Y, Yang F, Zhao H, Gao Q, Xia B, Fu J (2016). Research on the printability of hydrogels in 3D bioprinting. Sci Rep..

[5] Frontini López YR, Gojanovich AD, Masone DF, Bustos DM, Uhart M (2018). Adipose-derived mesenchymal stem/stromal cells: from the lab bench to the basic concepts for clinical translation. BioCell.

[6] Rivero G, Aldana AA, Lopez YF, Liverani L, Boccacini AR, Bustos DM (2019). 14-3-3ε protein-immobilized PCL-HA electrospun scaffolds with enhanced osteogenicity. J Mater Sci: Mater Med.

[7] Kaplan A, Bueno M, Fournier AE (2017). Extracellular functions of 14-3-3 adaptor proteins. Cell Signal.

[8] Millerand M, Sudre L, Nefla M, Pène F, Rousseau C, Pons A, Ravat A, André-Leroux G, Akira S, Satoh T, Berenbaum F, Jacques C (2020). Activation of innate immunity by 14-3-3 ε, a new potential alarmin in osteoarthritis. Osteoarthr Cartil.

[9] Nefla M, Sudre L, Denat G, Priam S, Andre-Leroux G, Berenbaum F (2015). The pro-inflammatory cytokine 14-3-3ε is a ligand of CD13 in cartilage. J Cell Sci.

[10] Romagnoli C, Brandi ML (2014). Adipose mesenchymal stem cells in the field of bone tissue engineering. World J Stem Cells.

[11] Aldana AA, Malatto L, Rehman MA, Boccaccini AR, Abraham GA (2019). Fabrication of Gelatin Methacrylate (GelMA) Scaffolds with Nano-and Micro-Topographical and Morphological Features. Nanomaterials..

[12] Uhart M, Iglesias AA, Bustos DM (2011). Structurally constrained residues outside the binding motif are essential in the interaction of 14-3-3 and phosphorylated partner. J Mol Biol.

[13] Gojanovich AD, Gimenez MC, Masone D, Rodriguez TM, Dewey RA, Delgui LR (2018). Human adipose-derived mesenchymal stem/stromal cells handling protocols. Lipid droplets and proteins double-staining. Front Cell Developmental Biol.

[14] Hughes D, Mehmet H, eds. Cell proliferation and apoptosis. Taylor & Francis, London; 2004.

[15] Fedorovich NE, Alblas J, Hennink WE, Öner FC, Dhert WJ (2011). Organ printing: the future of bone regeneration?. Trends Biotechnol.

[16] Miron RJ, Zhang YF (2012). Osteoinduction: a review of old concepts with new standards. J Dent Res.

[17] Xiao Z, Camalier CE, Nagashima K, Chan KC, Lucas DA, Cruz MJ (2007). Analysis of the extracellular matrix vesicle proteome in mineralizing osteoblasts. J Cell Physiol.

